# Transient Ca^2+^ entry by plasmalogen-mediated activation of receptor potential cation channel promotes AMPK activity

**DOI:** 10.3389/fmolb.2022.1008626

**Published:** 2022-11-04

**Authors:** Masanori Honsho, Shiro Mawatari, Takehiko Fujino

**Affiliations:** ^1^ Department of Neuroinflammation and Brain Fatigue Science, Graduate School of Medical Sciences, Kyushu University, Fukuoka, Japan; ^2^ Institute of Rheological Functions of Food, Fukuoka, Japan

**Keywords:** plasmalogens, TRPC4, AMPK, Ca^2+^, hair bulb, hair follicle, hair growth

## Abstract

Ethanolamine-containing alkenyl ether glycerophospholipids, plasmalogens, are major cell membrane components of mammalian cells that activate membrane protein receptors such as ion transporters and G-protein coupled receptors. However, the mechanism by which plasmalogens modulate receptor function is unknown. Here, we found that exogenously added plasmalogens activate transient receptor potential cation channel subfamily C member 4 (TRPC4) to increase Ca^2+^ influx, followed by calcium/calmodulin-dependent protein kinase 2-mediated phosphorylation of AMP-activated protein kinase (AMPK). Upon topical application of plasmalogens to the skin of mice, AMPK activation was observed in TRPC4-expressing hair bulbs and hair follicles. Here, TRPC4 was co-localized with the leucine-rich repeat containing G protein-coupled receptor 5, a marker of hair-follicle stem cells, leading to hair growth. Collectively, this study indicates that plasmalogens could function as gate openers for TRPC4, followed by activating AMPK, which likely accelerates hair growth in mice.

## 1 Introduction

Membrane receptor proteins localized to the plasma membrane play essential roles in cell and organ systems. Cells respond to extracellular stimuli through cell surface receptors and activate the downstream signaling cascade. Cell surface receptors are grouped into G-protein coupled receptors (GPCRs), ionotropic receptors, and tyrosine kinase receptors (Nair et al., 2019). Dysregulation of cell surface receptor proteins is associated with several diseases, including cancer, cystic fibrosis, cardiac arrhythmia, diabetes, and neurological disorders (Landfield and Pitler, 1984; Pierce et al., 2002; Lengyel et al., 2007; Croft, 2009; Schöneberg and Liebscher, 2021).

Plasmalogens are a subclass of glycerophospholipids characterized by the presence of a vinyl-ether bond at the *sn*-1 position of glycerol and are an important component of cellular membranes in mammalian cells. Several *in vitro* studies support that plasmalogens act as antioxidants and reservoirs of lipid mediators (Zoeller et al., 1999; Murphy, 2001; Nagan and Zoeller, 2001; Braverman and Moser, 2012). Plasmalogens modulate the synthesis, transport, and efflux of cholesterols (Mandel et al., 1998; Munn et al., 2003; Mankidy et al., 2010; Honsho et al., 2015; Honsho and Fujiki, 2017b). Plasmalogens are also involved in the regulation of protein kinase B (AKT) in Schwan cells, mouse embryonic fibroblasts, and neurons (da Silva et al., 2014; Malheiro et al., 2019; da Silva et al., 2021). In addition, supplementation of plasmalogens activates signaling pathways mediated by AKT, ERK, and brain-derived neurotrophic factors in cultured cells (Hossain et al., 2016; Che et al., 2020) and mice (Hossain et al., 2022), giving rise to enhanced learning and memory (Gu et al., 2022; Hossain et al., 2022), suggesting a potential function of plasmalogens in the regulation of signaling pathways.

In the present study, we investigated the role of plasmalogens in activating cellular signaling pathways in primary human dermal fibroblasts and found that plasmalogens enhance the phosphorylation of AMP-activated protein kinase (AMPK) by stimulating the activity of transient receptor potential cation channel subfamily C member 4 (TRPC4) in fibroblasts and hair follicles.

## 2 Materials and methods

### 2.1 Cell culture and dsRNA transfection

Primary human dermal fibroblasts (HDF-a) were purchased from Science Cell Research Laboratories and maintained in Fibroblast Medium. Human hair outer root sheath cells (HHORSC) and human hair dermal papilla cells (HHDPC) were purchased from Science Cell Research Laboratories and maintained Mesenchymal Stem Cell Medium, respectively, according to the manufacturer’s instructions (Science Cell Research Laboratories). HeLa and SH-SY5Y cells were maintained in Dulbecco’s Modified Eagle Medium (Sigma) supplemented with 10% FBS (Sigma), 50 μg/ml penicillin, and 50 μg/ml streptomycin. All cell lines were cultured at 37 °C under 5% CO_2_.

siRNA-mediated knockdown was performed using predesigned MISSION siRNA (Sigma). Cells were harvested at 72 h after initial transfection using Lipofectamine 20,000 (Thermo Fisher Scientific).

### 2.2 Antibodies and reagents

Rabbit antibodies against phospho-AMPKα (Thr172), phospho-Erk1/2 (Thr202/Tyr204), phospho-AKT (Ser473), AMPK, ERK, and AKT were purchased from Cell Signaling. Rabbit antibodies against calcium/calmodulin-dependent protein kinase kinase 2, beta (CaMKKβ; Proteintech), Keratin 15 (K15; Sigma), LGR5 (Sigma), and TRPC4 (Sigma) and mouse antibodies against GAPDH (Santa Cruz) were used.

The following siRNAs were used: human *CAMKK2* (SASI_Hs01_00108816) and *TRPC4* (SASI_Hs01_00129366). Guanosine 5’-[β-thio]diphosphate (GDPβS) and Verapamil were purchased from Sigma-Aldrich. SKF96365 and ML204 were purchased from Tokyo Chemical Industry Co., Ltd. and MedChemExpress, respectively.

Scallop derived plasmalogens (PlsEtn) and chicken derived plasmalogens (cPlsEtn) were extracted and purified as described previously (Mawatari et al., 2020). The fatty acid composition of plasmalogens in PlsEtn and cPlsEtn were analyzed as described (Honsho et al., 2022) and shown in Table 1. PlsEtn and cPlsEtn dissolved in OPTI-MEM (Thermo Fisher Scientific) by sonication were added at a final concentration of 5 μg/ml or 0.5 μg/ml in the medium of cells. Lyso-PlsEtn was prepared from hydrolysis of PlsEtn with honey bee venom PLA_2_ (Sigma) in Tris-HCl buffer (pH 8.5) containing 0.4 M CaCl_2_ and extracted by the Bligh and Dyer method (Bligh and Dyer, 1959). Lyso-PlsEtn dissolved in OPTI-MEM was added at a final concentration at 0.3 μg/ml in the medium of cells.

**TABLE 1:** Fatty acid composition of plasmalogens purified from scallop (PlsEtn) and chicken (cPlsEtn).

Fatty acid	PlsEtn (% w/w)	cPsEtn (% w/w)
linoleic acid	0.1	4.2
oleic acid	2.7	35.8
Eicosapentaenoic acid, EPA	24.3	2.0
Arachidonic acid	24.4	19.0
Docosahexaenoic acid, DHA	33.1	19.3
Other acids	15.4	19.7
Total	100.0	100.0

### 2.3 Immunoblot analysis

Cells were cultured in the presence of PlsEtn for the indicated time periods, harvested in homogenizing buffer, and then sonicated (Honsho and Fujiki, 2017a). Equal aliquots of total protein (3–10 μg) were separated using SDS-PAGE and transferred to a nitrocellulose membrane. The membrane was blocked by 1% BSA in TBST (10 mM Tris-HCl, pH 7.4, 200 mM NaCl, 0.05% Tween-20) for 1 h. The membrane was then incubated with primary antibodies overnight at 4°C, followed by incubation with secondary antibodies for 2 h at room temperature. Immunoblots were developed with Clarity™ Western ECL Substrate (Bio-Rad) and scanned with an ImageQuant LAS 4010 imager (GE Healthcare). The intensity of bands was quantified using the ImageJ software.

### 2.4 Calcium imaging

Intracellular calcium concentration of HDF-a cells was monitored using Fluo-4 AM (Dojindo laboratories) on a Keyence BZ-X800 fluorescent microscope (Keyence) at room temperature. Briefly, HDF-a cells were grown in a glass-bottom dish (Matsunami Glass Ind. Ltd.) for 18 h. The cells were washed with buffer A (20 mM Hepes, pH 7.4, 115 mM NaCl, 5.4 mM KCl, 0.8 mM MgCl_2_, 1 mM CaCl_2_, 13.8 mM Glucose) and further incubated for 1 h with 5 μg/ml Fluo-4 AM-containing buffer A in the presence or absence of inhibitors. The fluorophore was then rinsed with buffer A, followed by incubation at room temperature to thermodynamically equilibrate for 15 min. Images were acquired every 30 s. PlsEtn dissolved in OPTI-MEM (Thermo Fisher Scientific) by sonication were added with a pipette at a final concentration of 5 μg/ml 195 s after the beginning of each experiment.

### 2.5 Analyses of mouse hair growth and skin

Seven-year-old C3H/HeN mice were obtained from KBT Oriental Co., Ltd. and allowed to adapt for one week with free access to food and water. At eight weeks of age, dorsal skin hairs were removed with an animal clipper and wax (Epilae, Kracie). After two days, 20 μL of vehicle control or PlsEtn-containing solution prepared by suspension in water using sonication was topically applied daily for five days a week. Mice were sacrificed two weeks later. Dorsal skin tissues were obtained and fixed with 4% paraformaldehyde overnight. All experiments with live animals were approved by the Ethical Review Committee of the Institute of Rheological Functions of Food in accordance with the Guiding Principles for the Care and Use of Animals of the Physiological Society of Japan.

### 2.6 Histological analysis

The fixed tissues were dehydrated for embedding in paraffin. Five-micrometer tissue sections were subjected to either hematoxylin/eosin staining or immunohistochemical study using an Opal 4-Color Multiplex IHC kit, according to the manufacturer’s instructions (Akoya Bioscience). Briefly, skin sections were deparaffinized, rehydrated, and refixed with 10% neutral buffered formalin prior to antigen retrieval by heating for 15 min in AR6 retrieval buffer (Akoya Biosciences). Next, the tissue sections were subjected to sequential staining procedures including blocking, binding of the primary antibody and HRP-labeled secondary antibody where HRP-labeled secondary antibody catalyzes activation of tyramide-fluorophore to form covalent bonds with the tyrosine residues present in close proximity of the protein of interest. The tissues were heated to remove the primary and secondary antibodies leaving the covalently bound fluorophores on the tissue, and another primary antibody was used for the next step of staining.

### 2.7 Quantification and statistical analysis

The number of replicates, statistical significance, and number of animals are indicated in Figures or Figure legends. Data represent biological replicates. Statistical significance was determined by Student’s *t*-test, Wilcoxon rank sum test, or Chi-square test using the Excel software, or either Dunnett’s post-hoc test or Tukey–Kramer multiple comparisons test using the R software. All results are presented as mean ± standard deviation. Statistical significance was determined at *p <0.05 and **p <0.01.

## 3 Results

### 3.1 Effects of PlsEtn on cellular signaling pathways

**FIGURE 1:**
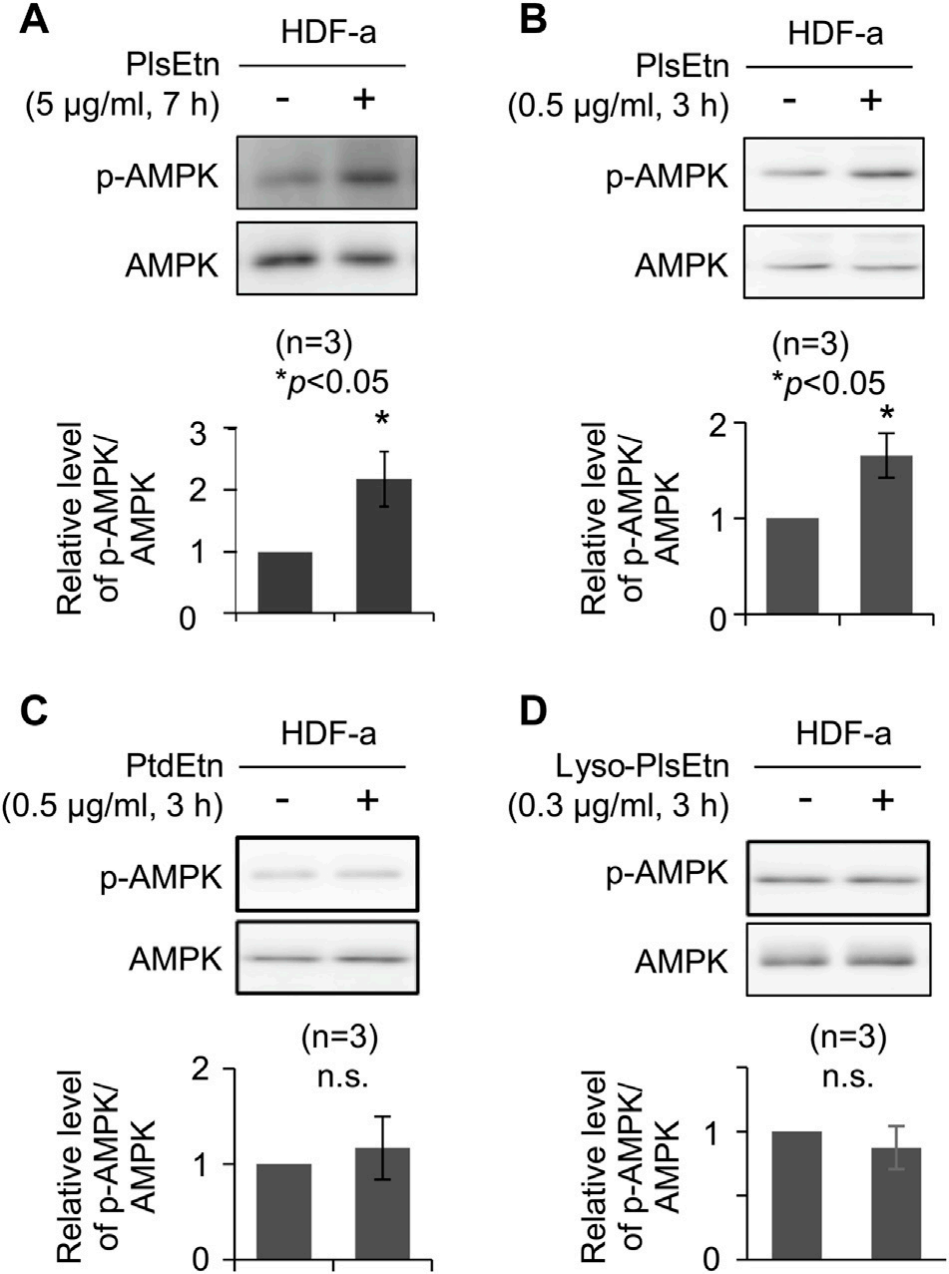
PlsEtn promote phosphorylation of AMPK in HDF-a cells **(A,B)** Immunoblot for phosphorylated AMPK (p-AMPK) and total AMPK in HDF-a cells cultured in the presence of 5 μg/ml **(A)** or 0.5 μg/ml **(B)** PlsEtn. The amount of p-AMPK to total AMPK is represented in the lower panel (n = 3) **(C,D)** Representative immunoblot for p-AMPK and AMPK in HDF-a cells treated with phosphatidylethanolamine (PtdEtn) **(C)** or 0.3 μg/ml lyso-plasmalogens (Lyso-PlsEtn) **(D)** Amount of p-AMPK to total AMPK is represented in the lower panel (*n* = 3).

We investigated the potential function of plasmalogens in activating cellular signaling pathways. To this end, the stimulation of cellular signaling pathways was verified in HDF-a. HDF-a cells were cultured in the presence of PlsEtn purified from scallop, followed by assessing the phosphorylation status of proteins involved in signaling pathways. We found that phosphorylation of AMPK at threonine 172 was significantly upregulated in the presence of PlsEtn (Figure 1A). However, neither the phosphorylation of ERK1/2 nor protein kinase B (PKB/AKT) at serine 473 were stimulated upon PlsEtn addition (Supplementary Figure S1), suggesting that PlsEtn likely enhance phosphorylation of AMPK in a manner independent of ERK and AKT. PlsEtn-mediated AMPK phosphorylation was enhanced in other human cells, such as HHDPC, HHORSC, neuroblastoma SH-SY5Y, and cervical cancer HeLa cells (Supplementary Figure S2), indicating that PlsEtn-mediated phosphorylation of AMPK is not a peculiarity of human dermal fibroblasts, but rather a general phenomenon.

Phosphorylation of AMPK was enhanced in the presence of a lower PlsEtn concentration (0.5 μg/ml) for a short incubation time (Figure 1B). We next investigated the structural requirements of plasmalogens toward the enhancement of AMPK phosphorylation. To this end, HDF-a cells were cultured in the presence of either lyso-PlsEtn prepared by enzymatic digestion of PlsEtn with phospholipase A_2_ or phosphatidylethanolamine (PtdEtn). We found that both phospholipids failed to stimulate phosphorylation of AMPK (Figures 1C,D). Taken together, these results suggest that the intact form of PlsEtn is required for the enhancement of AMPK phosphorylation.

### 3.2 PlsEtn enhance phosphorylation of AMPK in a CaMKKβ-dependent manner

**FIGURE 2:**
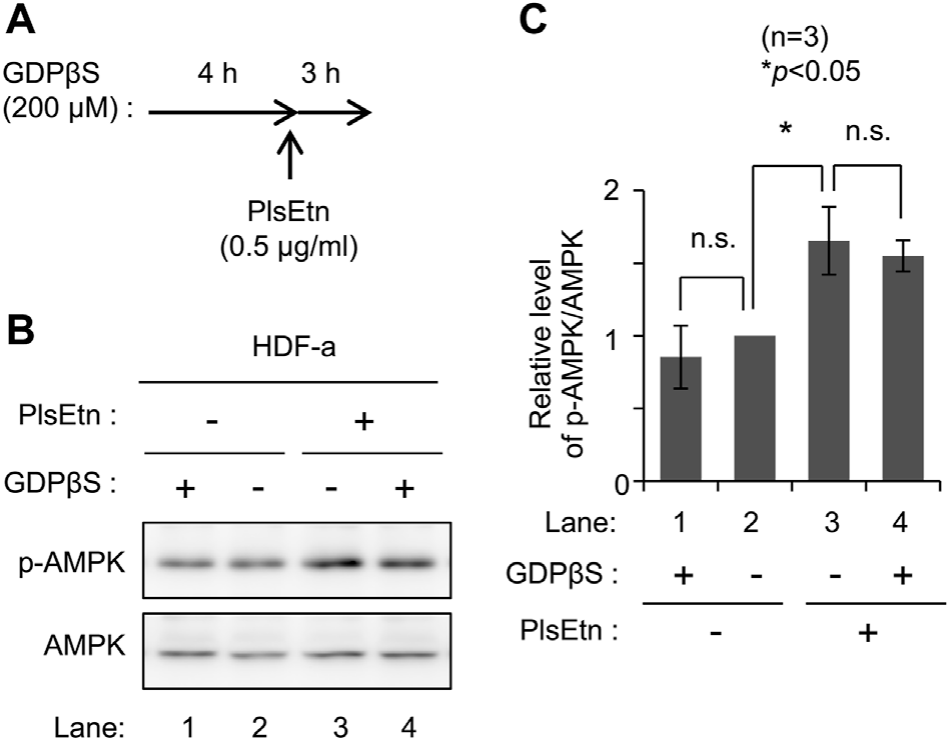
PlsEtn-mediated phosphorylation of AMPK is not inhibited by GDPβS **(A)** Schematic representation of the experimental procedure **(B)** Immunoblot for p-AMPK and AMPK in PlsEtn-treated HDF-a cells, cultured in the presence or absence of GDPβS **(C)** Amount of p-AMPK to total AMPK (*n* = 3). Statistical significance was determined by Tukey–Kramer multiple comparisons test using the R software.

To determine the molecular mechanisms underlying PlsEtn-mediated AMPK phosphorylation, we verified the involvement of G-protein coupled receptors in PlsEtn-mediated phosphorylation of AMPK. GPCRs can affect AMPK activity (Hutchinson et al., 2008). PlsEtn have been proposed to act as a ligand of GPCRs in a mouse Neuro-2a cell line (Hossain et al., 2016) and cultured bovine anterior pituitary cells (Kereilwe et al., 2018). Based on these notions, we investigated the involvement of GPCRs in PlsEtn-mediated AMPK phosphorylation in the presence of GDPβS, a nonhydrolyzable guanosine 5′-diphosphate analogue (Ma et al., 1994). Incubation of HDF-a cells with GDPβS did not impact AMPK phosphorylation in the presence or absence of PlsEtn (Figure 2), suggesting that PlsEtn-mediated activation of GPCRs is less likely to be involved in the increase in AMPK phosphorylation in HDF-a cells.

**FIGURE 3:**
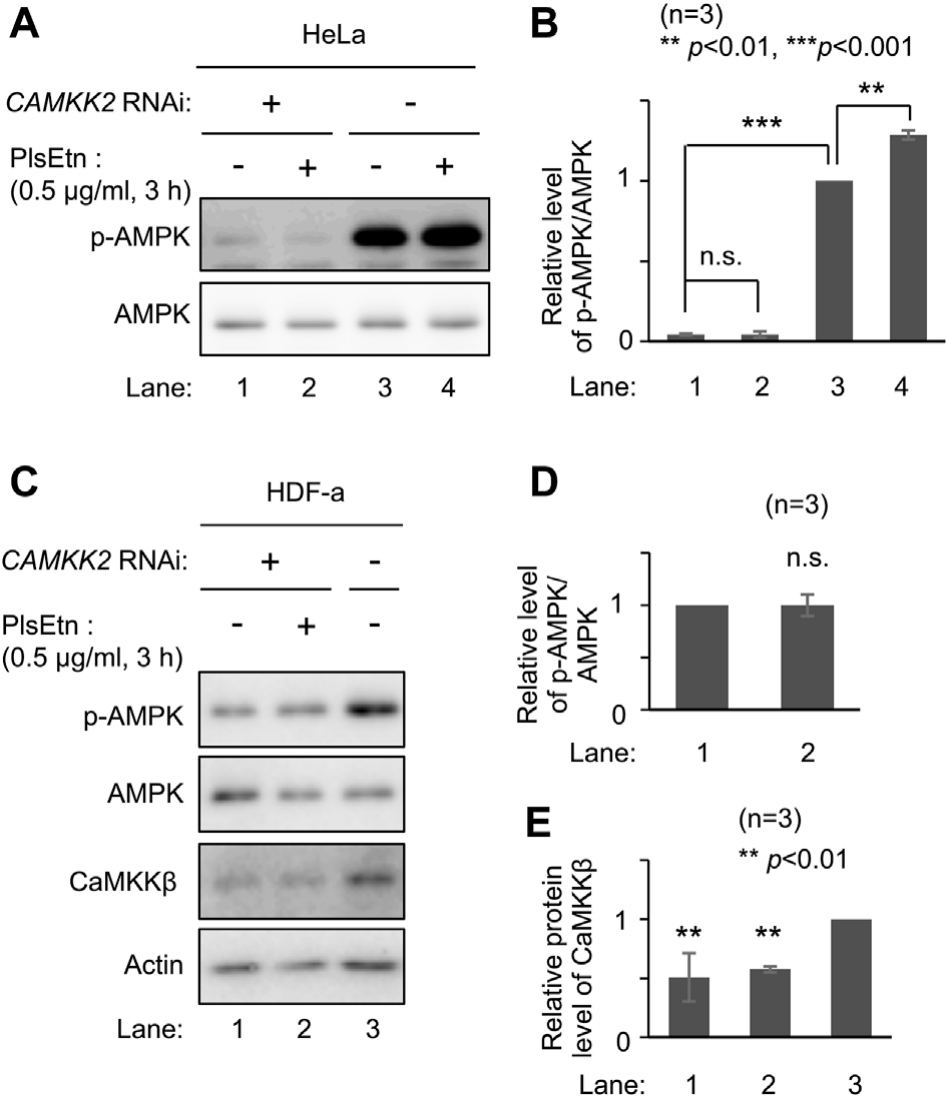
PlsEtn-mediated phosphorylation of AMPK is dependent on CaMKKβ **(A)** Immunoblot for p-AMPK and total AMPK in HeLa cells transfected with control dsRNA (-) or dsRNA against *CAMKK2* **(B)** Amount of p-AMPK to total AMPK (*n* = 3). Statistical significance was determined by Tukey–Kramer multiple comparisons test using the R software **(C)** Immunoblot for p-AMPK and total AMPK in HDF-a cells transfected with control dsRNA (-) or dsRNA against *CAMKK2* **(D)** Amount of p-AMPK to total AMPK in **(C)** (*n* = 3) **(E)** Protein level of CaMKKβ normalized to that of actin (*n* = 3).

AMPK is phosphorylated by upstream kinases, liver kinase B1 (LKB1) (Hawley et al., 2003; Woods et al., 2003; Shaw et al., 2004) and CaMKKβ (Hawley et al., 2005; Hurley et al., 2005; Woods et al., 2005). We attempted to identify the upstream kinase in PlsEtn-mediated phosphorylation of AMPK. We selected HeLa cells because LKB1 is not expressed in this line (Hawley et al., 2003). Phosphorylation of AMPK was enhanced upon culturing cells in the presence of PlsEtn (Figure 3A; Supplementary Figure S2). Contrary to this, transfection of double-stranded RNA against *CAMKK2* encoding CaMKKβ dramatically reduced phosphorylation of AMPK and failed to enhance phosphorylation even in the presence of PlsEtn (Figures 3A,B). We further verified the involvement of CaMKKβ in plasmalogen-mediated phosphorylation of AMPK by reducing its expression in HDF-a cells. The level of phosphorylated AMPK was markedly decreased by lowering CaMKKβ expression (Figures 3C,E). Under the same conditions, the phosphorylated AMPK level was not augmented in the presence of PlsEtn (Figures 3C,D). Taken together, these results suggest that plasmalogens enhance phosphorylation of AMPK in a CaMKKβ-dependent manner.

### 3.3 Increased intracellular Ca^2+^ concentration by PlsEtn

**FIGURE 4:**
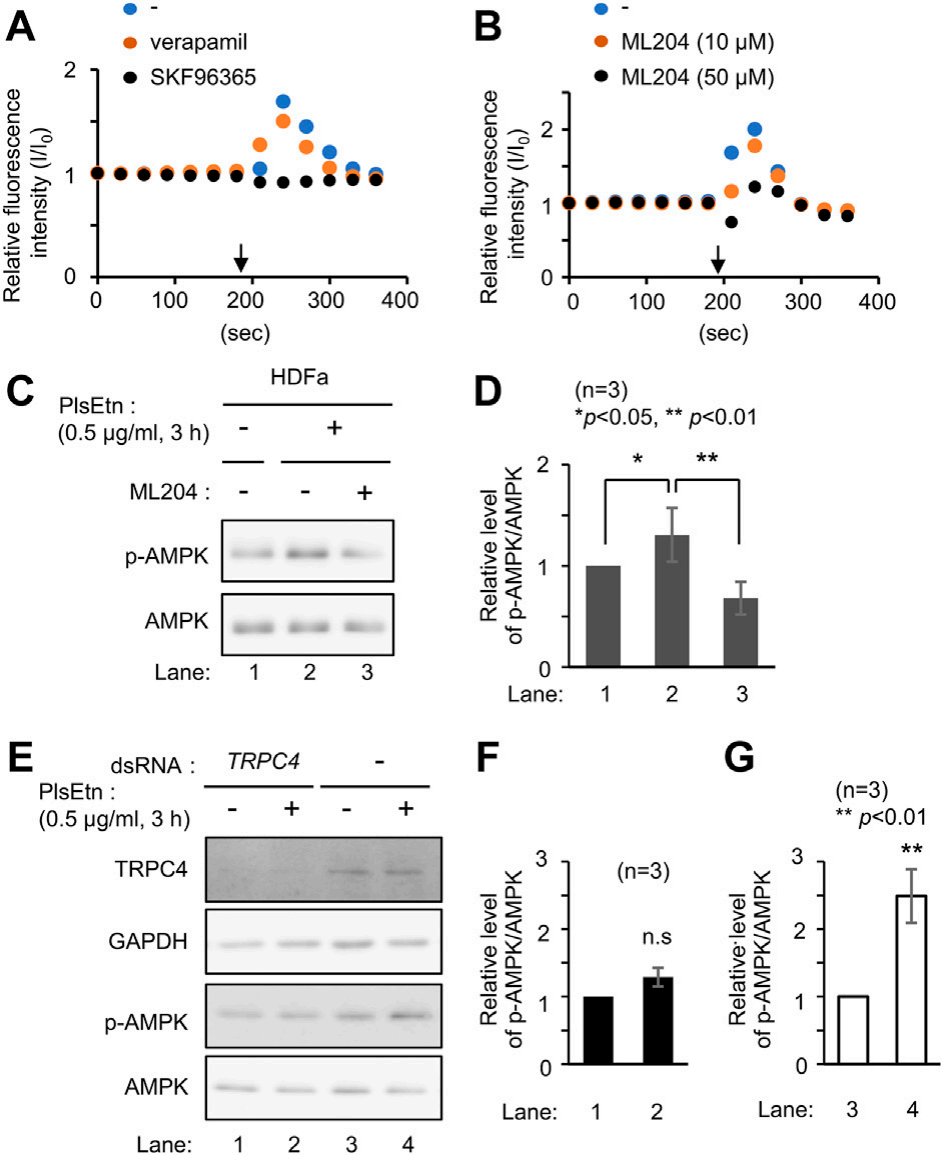
PlsEtn activate TRPC4, leading to enhanced phosphorylation of AMPK **(A)** Intracellular Ca^2+^ concentration of HDF-a cells loaded with Fluo-4 AM before and after PlsEtn stimulation (arrow) in the presence of vehicle (-), verapamil, or SKF96365. Fluorescence intensity at each time point normalized to that at time 0 was calculated and plotted **(B)** Fluorescence intensity of PlsEtn-stimulated HDF-a cells in the presence of vehicle (-) or ML204 **(C)** Immunoblot for p-AMPK and total AMPK in untreated HDF-a cells (-) or cells treated with PlsEtn in the absence (-) or presence (+) of ML204 (50 μM) for 7 h **(D)** Amount of p-AMPK to total AMPK in **(C)** (*n* = 3) **(E)** Immunoblot for p-AMPK and total AMPK in PlsEtn-unstimulated (-) or -stimulated (+) HDF-a cells transfected with control dsRNA or dsRNA against *TRPC4* **(F,G)** Amount of p-AMPK to total AMPK in **(E)** (*n* = 3).

Increasing intracellular Ca^2+^ leads to AMPK activation in a CaMKKβ-dependent manner (Hurley et al., 2005; Woods et al., 2005; Hardie, 2008). Therefore, we investigated the potential function of plasmalogens in calcium influx by monitoring intracellular Ca^2+^ levels using a fluorescent calcium indicator Fluo-4 AM. Ca^2+^ level was elevated immediately after adding PlsEtn to the medium, suggesting that plasmalogens likely affect the activity of plasma membrane-localized channel(s) (Figures 4A,B).

Extracellular Ca^2+^ influx across the plasma membrane is mediated by different types of channels, including transient receptor potential channels (Venkatachalam and Montell, 2007), and voltage-gated Ca^2+^ channels (Catterall, 2011). To identify the channel activated by plasmalogens, cells were treated with channel inhibitors, followed by analysis of intracellular Ca^2+^ levels by adding PlsEtn in the presence of inhibitors. The L-type voltage-gated calcium channel blocker verapamil (Zhao et al., 2019) failed to suppress the influx of Ca^2+^ upon addition of PlsEtn (Figure 4A). Contrary to this, Ca^2+^ influx was completely abolished in the presence of SKF96365, an inhibitor extensively used to define the role of the canonical transient receptor potential (TRPC) channel (Bengtson et al., 2004; Chae et al., 2012) (Figure 4A). Among the five TRPC channels expressed in humans, TRPC3 and TRPC6 are directly activated by diacylglycerol (Hofmann et al., 1999); however, the activation of the remaining TRPC channels is not well understood. To investigate the involvement of the remaining three TRPC channels, we chose ML204, a selective TRPC4 channel inhibitor (Miller et al., 2011). We observed that PlsEtn-mediated Ca^2+^ influx was strongly suppressed in the presence of ML204, suggesting that the influx of Ca^2+^ is caused by PlsEtn-mediated activation of TRPC4 (Figure 4B). Consistent with these results, PlsEtn-mediated phosphorylation of AMPK was diminished in the presence of ML204 or knockdown of TRPC4 (Figures 4C–G). Thus, these results indicate that PlsEtn enhance phosphorylation of AMPK through activation of TRPC4. A slight stimulation of a transient Ca^2+^ influx in HDF-a cells was observed when cPlsEtn was used for activation of TRPC4 (Supplementary Figure S3). Consistent with this observation, cPlsEtn failed to activate AMPK (Supplementary Figure S3). PlsEtn are enriched in polyunsaturated fatty acids such as docosahexaenoic acids (DHA), whereas cPlsEtn containing monosaturated fatty acid (oleic acid) (Table 1). These results suggest that PUFA-containing PlsEtn likely act as a gate opener for TRPC4.

### 3.4 PlsEtn-mediated activation of AMPK in skin

Activation of AMPK by 5-aminoimidazole-4-carboxamide ribonucleotide, an AMP analog, or metformin, agonist of AMPK, induces hair regeneration by promoting hair follicle growth stage (anagen) entry from the resting stage (telogen) (Chai et al., 2019). Plasmalogens secreted from keratinocytes are involved in pathological conditions such as psoriasis and skin cancer (Yamamoto et al., 2015). Based on these findings, we evaluated whether plasmalogens stimulate hair growth through activation of AMPK. In mammals, hair grows in a repetitive cycle with telogen, anagen, and a regression stage (catagen) (Müller-Röver et al., 2001; Schneider et al., 2009).

**FIGURE 5:**
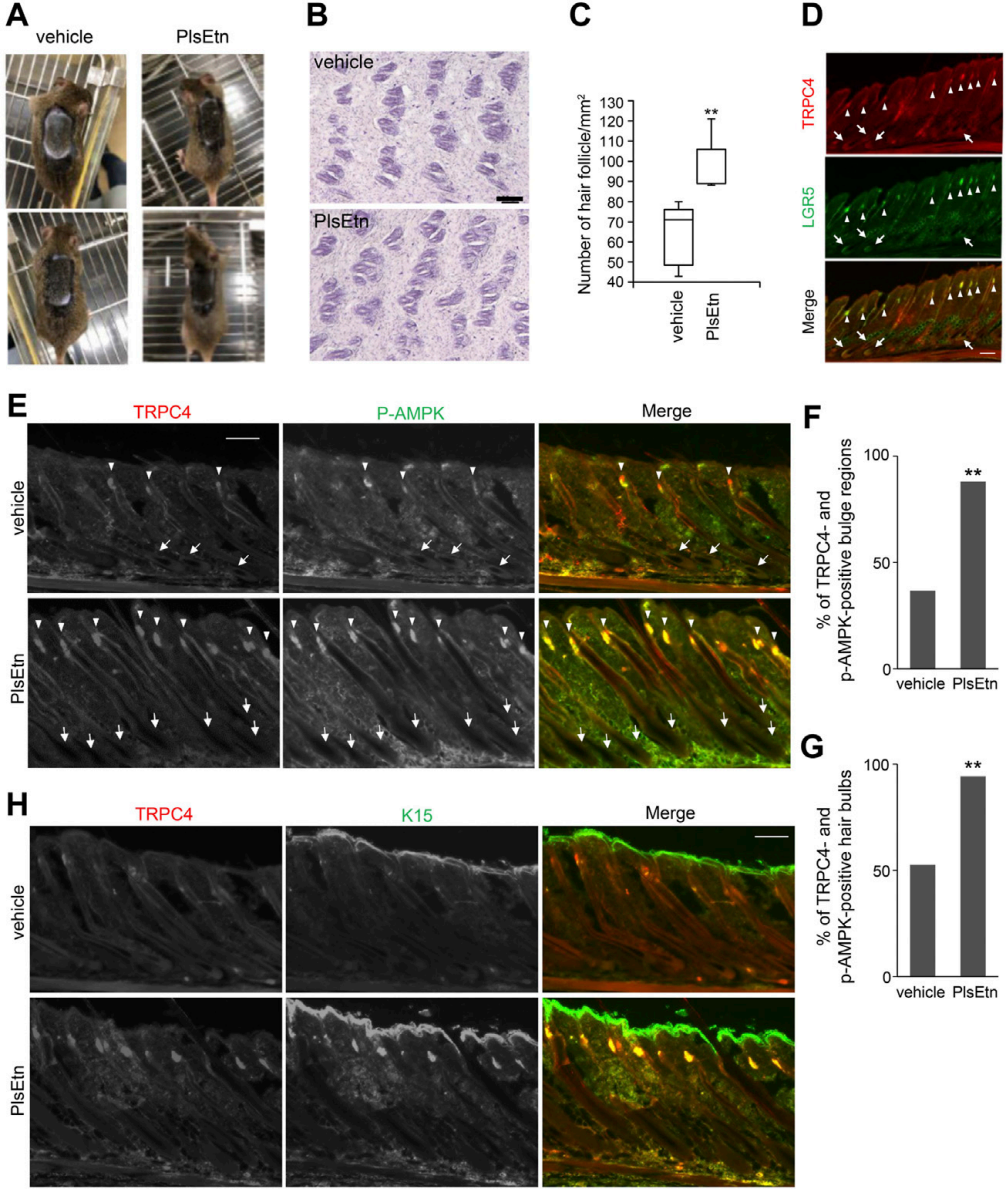
PlsEtn activate cells in bulge and hair bulb, leading to hair growth stimulation **(A)** PlsEtn induce hair regeneration. Eight-week-old male C3H/HeN mice were shaved and topically applied with vehicle (left) or 10 mg/ml PlsEtn (right) daily for five days a week. Photographs were taken on day 14 post-treatment. Number of animals: vehicle, *n* = 5; PlsEtn, *n* = 3. Interestingly, 10 mg/ml PlsEtn generated similar results to 5 mg/ml (Supplementary Figure S4) **(B)** Hematoxylin and eosin staining in dorsal skin treated with vehicle or PlsEtn (10 mg/ml) **(C)** Number of hair follicles per mm^2^ counted in five skins from mice treated with vehicle (*n* = 2) and PlsEtn (*n* = 2), respectively. Statistical analysis was performed by Wilcoxon rank sum test **(D)** Localization of TRPC4 and LGR5 in unshaved dorsal skin. The hair bulges and hair bulbs where TRPC4 and LGR5 localize are indicated by arrow heads and arrows, respectively **(E)** Immunofluorescence signals of p-AMPK and TRPC4 in dorsal skin treated with vehicle (upper panels) or 5 mg/ml PlsEtn (lower panels). TRPC4- and p-AMPK-positive hair bulbs and bulges are indicated by arrows and arrow heads, respectively **(F)** Percentages of TRPC4- and p-AMPK-positive bulge in dorsal skin of mice treated with vehicle (*n* = 3) or 5 mg/ml PlsEtn (*n* = 3). More than 200 hair bulges were randomly counted in each condition. Statistical analysis was performed by χ^2^ test **(G)** Percentages of TRPC4- and p-AMPK-positive hair bulbs in the dorsal skin of mice treated with vehicle (*n* = 3) or 5 mg/ml PlsEtn (*n* = 3). More than 200 hair bulbs were randomly counted in each condition. Statistical analysis was performed by χ^2^ test **(H)** Immunofluorescence signals of TRPC4 and K15 in dorsal skin treated with vehicle (upper panels) or 5 mg/ml PlsEtn (lower panels).

Dorsal skin hairs in the telogen phase (Müller-Röver et al., 2001) were removed from eight-week-old male C3H/HeN mice followed by topical application of PlsEtn or vehicle control 48 h later, daily for five days a week. Hair regeneration was enhanced by PlsEtn at the concentrations used (5 and 10 mg/ml) compared with vehicle treatment (Figure 5A; Supplementary Figure S4). When horizontally sliced skin was stained with hematoxylin and eosin, hair follicles appeared to increase in number in PlsEtn-treated dorsal skin compared to the control (Figures 5B,C). These results suggest that plasmalogens induce telogen to anagen transition and hair follicle growth.

To assess whether PlsEtn-induced hair regeneration is associated with expression of TRPC4, we investigated the expression of TRPC4 in murine dorsal skin by staining with antibodies against TRPC4 and LGR5, which are expressed in bulges and the outer root sheath during anagen (Hsu et al., 2011). TRPC4 expression was observed in hair bulbs and hair follicles underneath the skin, and TRPC4 was co-localized with LGR5, implying that TRPC4 is expressed in cells located in bulges in addition to hair bulbs (Figure 5D).

Next we verified whether PlsEtn augment the phosphorylation of AMPK in TRPC4 localized hair bulbs and hair follicles by staining with antibodies against TRPC4 and p-AMPK (Figure 5E). In PlsEtn-treated skin, cells in hair bulbs and bulges were mostly positive with both TRPC4 and p-AMPK antibodies (Figures 5E–G). Contrary to this, hair bulbs with TRPC4- and p-AMPK-positive signals were less frequently observed in vehicle-treated dorsal skin (Figures 5E–G). These results suggest that plasmalogens promote the activation of AMPK in a TRPC4-dependent manner. We further assessed K15, which is specifically expressed in human scalp hair follicle bulges and preferentially expressed in adult mouse bulge stem cells (Lyle et al., 1998, 1999; Liu et al., 2003). These bulge stem cells proliferate during the anagen phase of the hair cycle and rapidly divide at the onset of new hair follicle growth (Lyle et al., 1998). In vehicle-treated dorsal skin, K15 was observed in the bulges of the hair follicle together with TRPC4, whereas only K15 was observed in the epidermis, as reported previously (Porter et al., 2000; Tu et al., 2005). The localization of K15 and TRPC4 in the bulge of hair follicles became readily discernible upon PlsEtn treatment (Figure 5H). Collectively, these results suggest that PlsEtn activate AMPK in TRPC4 localized hair bulbs and hair follicles, resulting in hair regeneration by promoting stem cell activity in the bulge of hair follicles.

## 4 Discussion

Plasmalogens are major structural glycerophospholipids in the cell membrane and are involved in many processes including membrane fusion and ion transport. Plasmalogens increase the activity of sodium-calcium exchangers predominantly located in the plasma membrane (Ford and Hale, 1996). In this study, we demonstrated that PlsEtn enhance Ca^2+^ influx by activating TRPC4, followed by phosphorylation of AMPK in a CaMKKβ-dependent manner. The involvement of TRPC4 in PlsEtn-mediated Ca^2+^ influx was further supported by the findings that the TRPC4 channel inhibitor ML204 strongly diminished Ca^2+^ influx and AMPK phosphorylation (Figure 4). Neither PtdEtn nor lyso-PlsEtn promoted AMPK activation, suggesting that the vinyl-ether bond at the *sn*-1 position of plasmalogens together with fatty acid moiety at *sn*-2 position is required for the activation of TRPC4.

**FIGURE 6:**
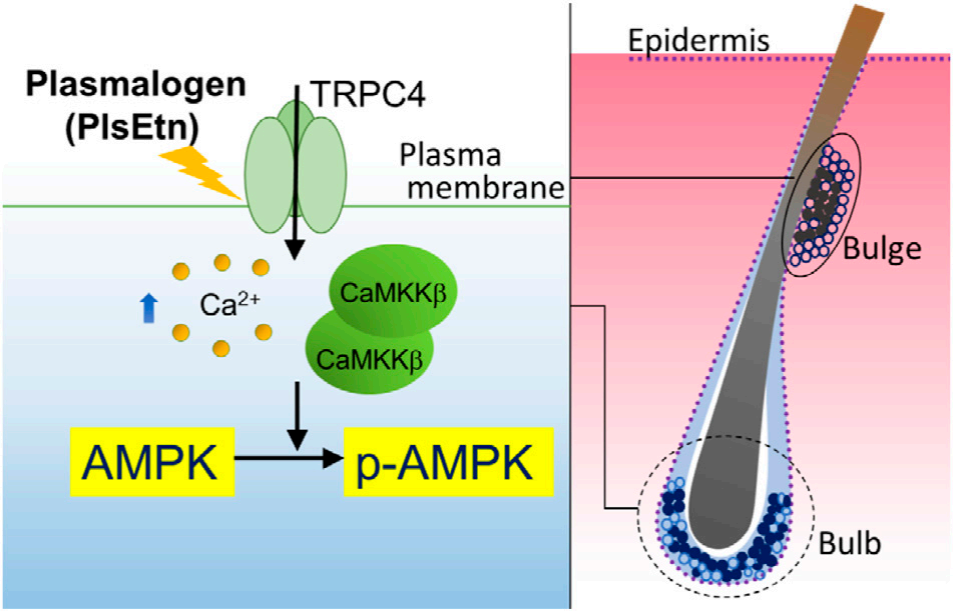
Proposed model of PlsEtn-mediated activation of AMPK in cells (left), hair bulge, and hair bulb (right). PlsEtn activate AMPK by enhancing transient Ca^2+^ entry *via* modulation of TRPC4 channel activity in cells including HDF-a, HHDPC, and HHORSC. In stem cells of hair bulges and follicles (solid circle), PlsEtn likely induce AMPK activation *via* upregulation of TRPC4 channel activity, giving rise to enhance hair growth.

Plasmalogen-mediated activation of GRCRs, such as GPR61, was demonstrated in cultured neuronal cells (Hossain et al., 2016). A recent study revealed the enhanced secretion of follicle-stimulating hormone by plasmalogens but not lyso-plasmalogens in cultured bovine anterior pituitary cells. From these results, the authors postulated the role of plasmalogen as a ligand for GPR61 (Kereilwe et al., 2018). Here, we found that GDPβS failed to inhibit PlsEtn-mediated activation of AMPK in HDF-a cells (Figure 2). Furthermore, elevation of intracellular Ca^2+^ was quickly observed upon adding PlsEtn (Figures 4A,B). Based on these results, we assumed that PlsEtn directly activate TRPC4 in a manner independent of GPCRs (Figure 6). The finding that TRPC4 activation is promoted by PUFA-enriched PlsEtn, but not cPlsEtn which are enriched in oleic acid (Table 1), suggests that activation of TRPC4, i.e., transient opening of the narrow gate of TRPC4 to pass Ca^2+^ (Duan et al., 2018), is promoted by a ligand functions of PUFA-containing PlsEtn to TRPC4 or locally altering fluidity of plasma membrane. Clearly, further studies are required for elucidating the precise mechanism underlying the PlsEtn-mediated activation of TRPC4.

We observed the expression of TRPC4 in the epidermis of dorsal skin, consistent with the earlier study which showed TRPC4 expression in primary cultured keratinocytes from the epidermis (Figure 5) (Porter et al., 2000; Tu et al., 2005). In addition, the TRPC4-positive signal merged with LGR5 or K15 in the bulge of some hair follicles in the dorsal skin of untreated mice, where AMPK is phosphorylated (Figure 5). Striking elevation of TRPC4- and p-AMPK-positive signals appeared in most bulges and hair bulbs upon treatment with PlsEtn (Figure 5). PlsEtn likely enhance phosphorylation of AMPK through activation of TRPC4 as observed in HHORSC and HHDPC, mesenchymal cells isolated from hair dermal papilla cells (Supplementary Figure S2). Based on these findings, we propose that PlsEtn infiltrate hair follicles, leading to AMPK phosphorylation through the activation of TRPC4 in bulges and hair bulbs by mechanisms similar to those underlying plasmalogen-mediated activation of AMPK in HDF-a cells (Figure 6). Further elucidation of the role of PlsEtn in TRPC4 activation, including elevation of its expression, is required to understand the mechanism of PlsEtn-induced activation of AMPK in skin. AMPK is activated by caloric restriction, endurance exercise, resveratrol, and metformin (Martin-Montalvo et al., 2013; Weir et al., 2017; Steinberg and Carling, 2019). The mice model experiments demonstrated that caloric restriction enlarges the hair follicle stem cell pool and promotes increased hair follicle growth (Forni et al., 2017). Caloric restriction induces autophagy and this pharmacological activation triggers hair growth (Chai et al., 2019). Therefore, activation of AMPK is likely to be a promising method for stimulating hair growth. Indeed, a number of small molecules known to activate AMPK have the potential to induce hair growth in mice model experiments. For instance, anagen induction and hair regeneration are stimulated by topical treatment with AMPK-activating molecules, such as metformin, a widely used anti-diabetic agent (Chai et al., 2019), and resveratrol (Kubo et al., 2020). Metformin activates AMPK in an LKB1-dependent manner (Shaw et al., 2005) by reducing ATP production *via* inhibition of complex I in the mitochondrial respiratory chain (Owen et al., 2000; Hawley et al., 2010; Steinberg and Carling, 2019). Resveratrol inhibits ATP synthesis by binding ATP synthase (Zheng and Ramirez, 2000; Kipp and Ramirez, 2001; Gledhill et al., 2007) and elevates the AMP:ATP ratio, thereby activating AMPK in a LKB1-dependent manner (Lan et al., 2017). Unlike LKB1-mediated activation of AMPK, PlsEtn-mediated activation was observed in LKB1-deficient HeLa cells. This was strikingly diminished by reduction of CaMKKβ expression in both HeLa and HDF-a cells, implying that plasmalogens primarily stimulate phosphorylation of AMPK by enhancing Ca^2+^ influx followed by activation of CaMKKβ.

Plasmalogens can be found in almost all mammalian tissues (Braverman and Moser, 2012). In plasma membranes, plasmalogens are located in the inner leaflet due to the flippase activity of P4-ATPases, belonging to the superfamily of P-type ATPases (Honsho et al., 2017; Honsho et al., 2022). Therefore, these cellular plasmalogens located in the inner leaflet of plasma membrane are less likely to be involved in the activation of TRPC4. The physiological role of plasmalogen-mediated activation of TRPC4 is under investigation. Interestingly, plasmalogens secreted from keratinocytes and plasmalogens containing DHA are preferentially digested by group IIF-secreted phospholipase A_2_ under pathological conditions (Yamamoto et al., 2015). Therefore, in the skin, DHA-containing plasmalogens secreted from keratinocytes may have the potential to activate AMPK in hair follicles. Further studies on hair regeneration in plasmalogen-deficient mice will shed light on the physiological role of secreted plasmalogens in skin.

## Data Availability

The original contributions presented in the study are included in the article/Supplementary Materials, further inquiries can be directed to the corresponding author.
