# Supplementary material for: Transient Ca2+ entry by plasmalogen-mediated activation of receptor potential cation channel promotes AMPK activity
Source: Front Mol Biosci. 2022 Nov 4;9:1008626. doi: 10.3389/fmolb.2022.1008626 (PMC9672372; doi:10.3389/fmolb.2022.1008626)
Supplement: Supplementary file 1 [file Presentation1.PPTX]

## Slide 1
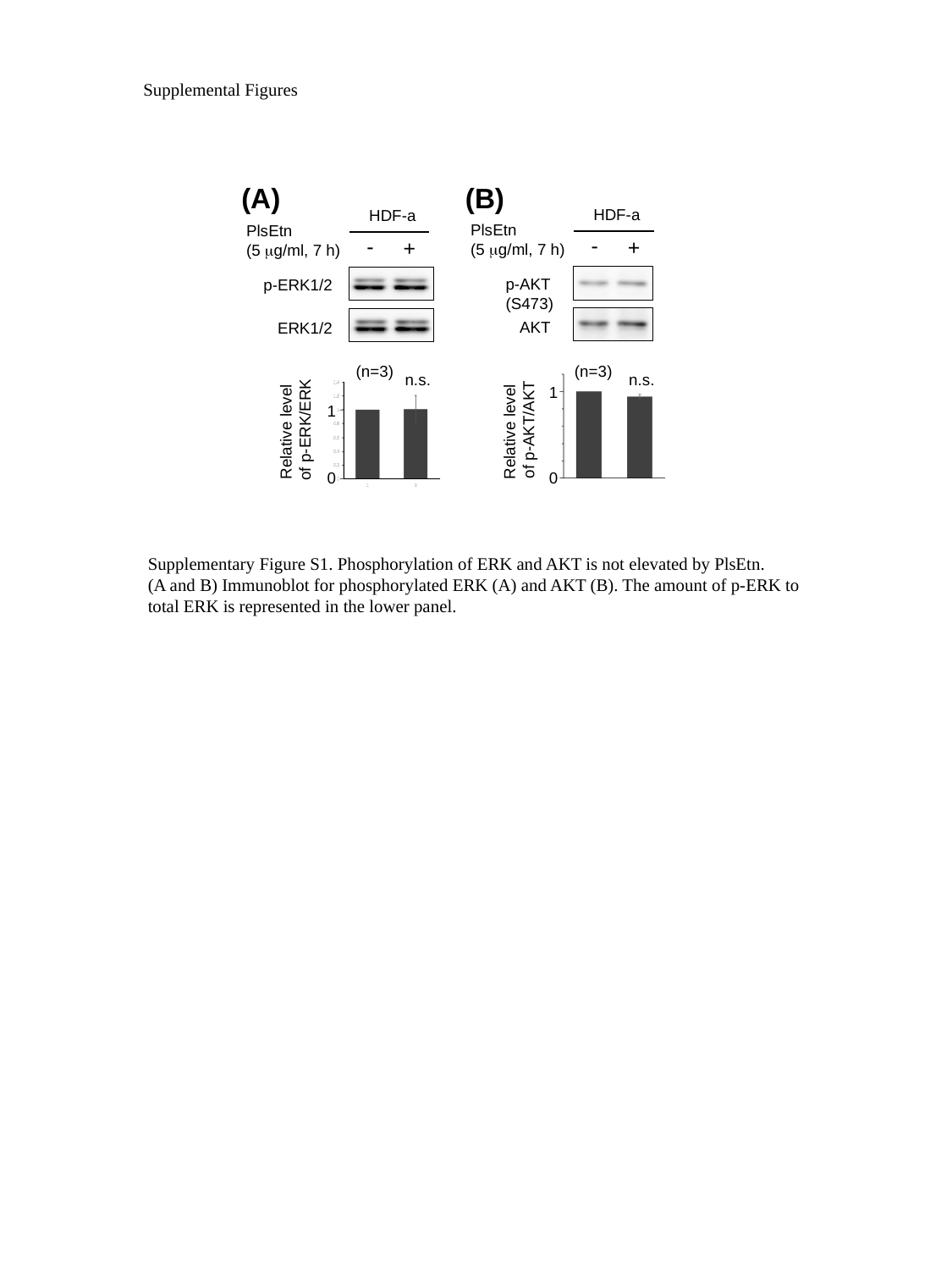

Supplemental Figures
(A)
(B)
HDF-a
HDF-a
PlsEtn
(5 mg/ml, 7 h)
PlsEtn
(5 mg/ml, 7 h)
-
-
+
+
p-AKT
(S473)
p-ERK1/2
AKT
ERK1/2
(n=3)
(n=3)
n.s.
n.s.
1
1
Relative level
of p-ERK/ERK
Relative level
of p-AKT/AKT
0
0
Supplementary Figure S1. Phosphorylation of ERK and AKT is not elevated by PlsEtn.
(A and B) Immunoblot for phosphorylated ERK (A) and AKT (B). The amount of p-ERK to
total ERK is represented in the lower panel.

## Slide 2
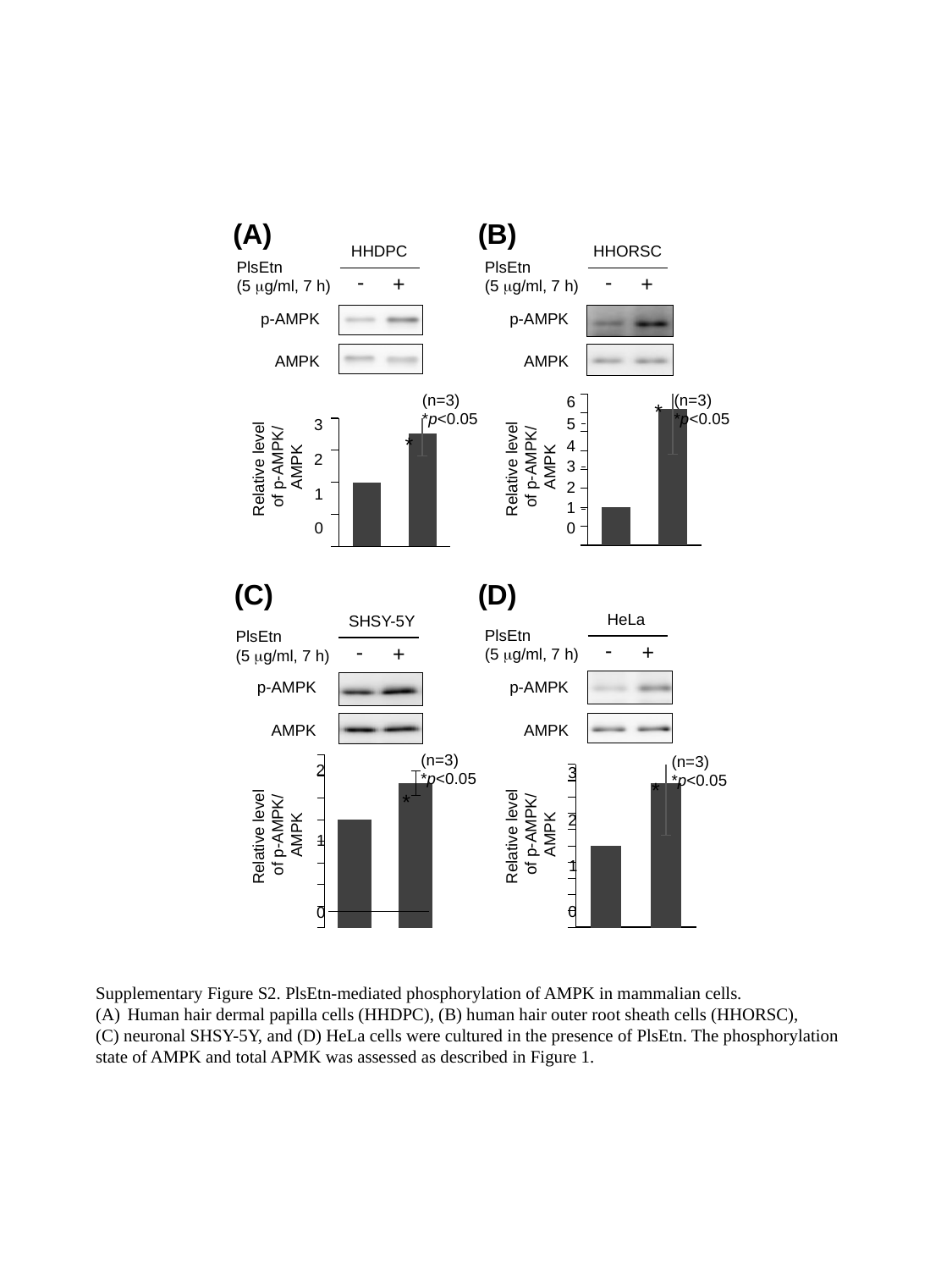

(A)
(B)
HHDPC
HHORSC
PlsEtn
(5 mg/ml, 7 h)
PlsEtn
(5 mg/ml, 7 h)
-
-
+
+
p-AMPK
p-AMPK
AMPK
AMPK
### Chart
| Category | |
|---|---|(n=3)
*p<0.05
(n=3)
*p<0.05
6
*
### Chart
| Category | |
|---|---|5
3
*
4
Relative level
of p-AMPK/
AMPK
Relative level
of p-AMPK/
AMPK
2
3
2
1
1
0
0
(C)
(D)
HeLa
SHSY-5Y
PlsEtn
(5 mg/ml, 7 h)
PlsEtn
(5 mg/ml, 7 h)
-
-
+
+
p-AMPK
p-AMPK
AMPK
AMPK
(n=3)
*p<0.05
(n=3)
*p<0.05
### Chart
| Category | |
|---|---|
### Chart
| Category | |
|---|---|2
3
*
*
Relative level
of p-AMPK/
AMPK
Relative level
of p-AMPK/
AMPK
2
1
1
0
0
-
Supplementary Figure S2. PlsEtn-mediated phosphorylation of AMPK in mammalian cells.
(A)	Human hair dermal papilla cells (HHDPC), (B) human hair outer root sheath cells (HHORSC),
(C) neuronal SHSY-5Y, and (D) HeLa cells were cultured in the presence of PlsEtn. The phosphorylation
state of AMPK and total APMK was assessed as described in Figure 1.

## Slide 3
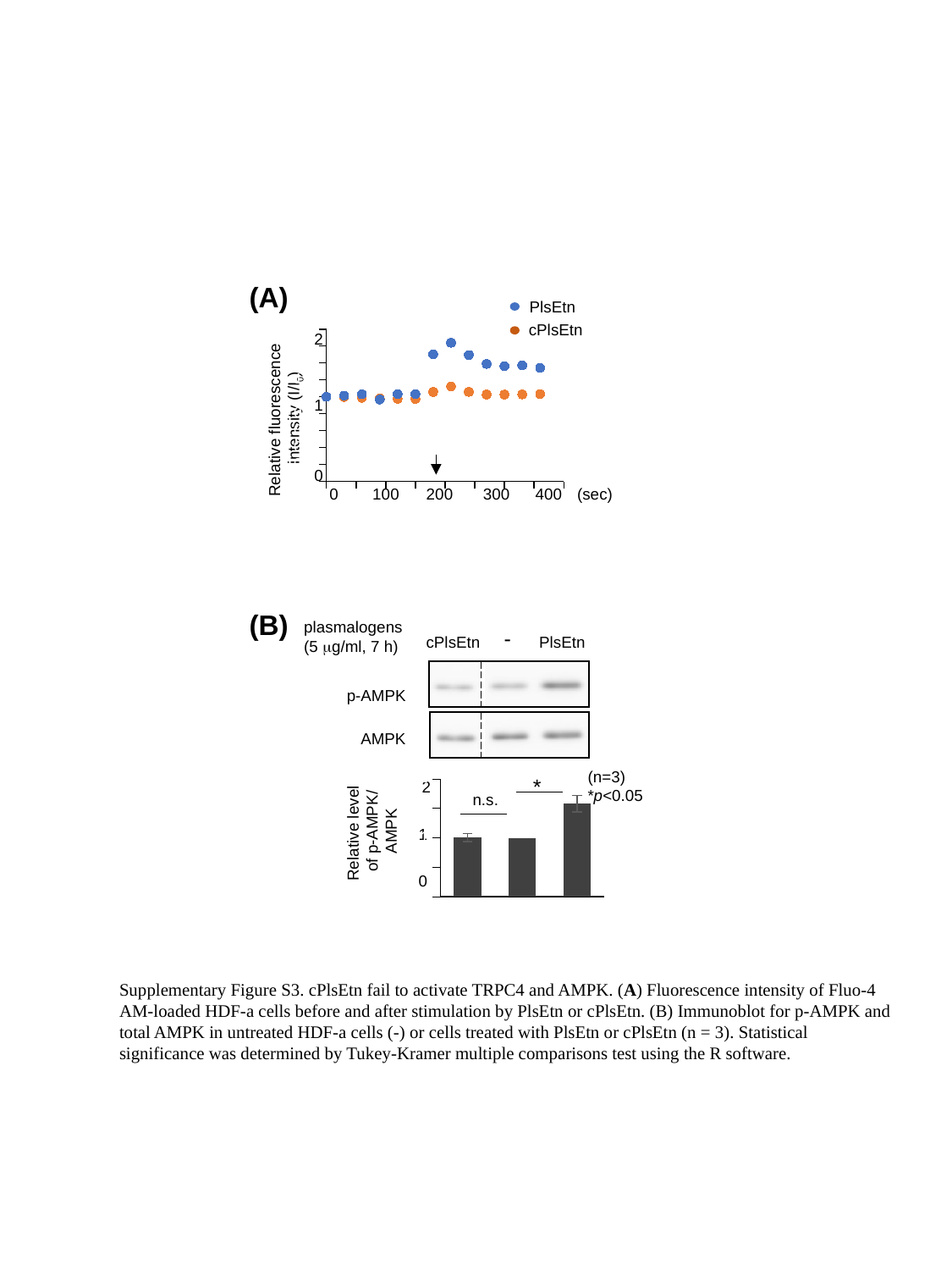

(A)
PlsEtn
cPlsEtn
### Chart
| Category | | |
|---|---|---|2
1
Relative fluorescence
 intensity (I/I0)
0
0
100
200
300
400
(sec)
(B)
plasmalogens
(5 mg/ml, 7 h)
-
cPlsEtn
PlsEtn
p-AMPK
AMPK
(n=3)
*p<0.05
*
### Chart
| Category | |
|---|---|2
n.s.
Relative level
of p-AMPK/
AMPK
1
0
Supplementary Figure S3. cPlsEtn fail to activate TRPC4 and AMPK. (A) Fluorescence intensity of Fluo-4 AM-loaded HDF-a cells before and after stimulation by PlsEtn or cPlsEtn. (B) Immunoblot for p-AMPK and total AMPK in untreated HDF-a cells (-) or cells treated with PlsEtn or cPlsEtn (n = 3). Statistical significance was determined by Tukey-Kramer multiple comparisons test using the R software.

## Slide 4
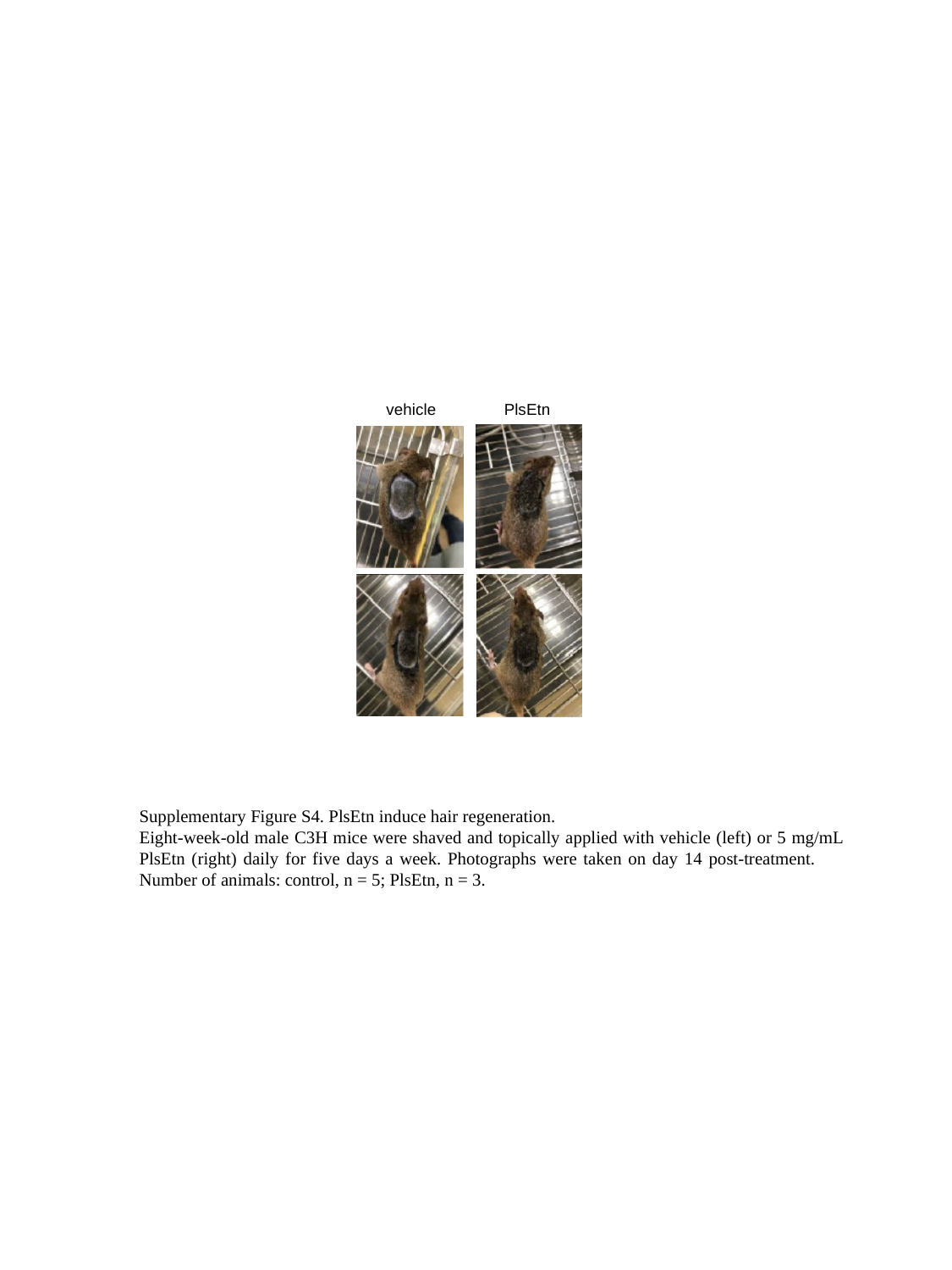

vehicle
PlsEtn
Supplementary Figure S4. PlsEtn induce hair regeneration.
Eight-week-old male C3H mice were shaved and topically applied with vehicle (left) or 5 mg/mL PlsEtn (right) daily for five days a week. Photographs were taken on day 14 post-treatment.　Number of animals: control, n = 5; PlsEtn, n = 3.
